# Some Observations Relative to the New Method of Extracting the Cataract, as Recently Proposed by Dr. Lobenstein Lobel in the Edinburgh Medical and Surgical Journal

**Published:** 1817-04

**Authors:** Edward Chapman


*For the London Medical and Physical Journal.*

### *Some Observations relative to the New Method of Extracting the Cataract, as recently proposed by Dr. Lobenstein Lobel in the Edinburgh Medical and Surgical Journal;* by Edward Chapman, Esq.

ABOUT six months prior to the date of this communication, on considering the many objections which admit of being urged against the operation of extracting the cataract through the aperture caused by a circular incision of the transparent cornea, an idea suggested itself to me, whether or not there existed a possibility of removing the opaque lens by means of an incision through the schlerotic and choroid coats, at a few lines distance from the junction of these tunics with the cornea itself.

In order to ascertain the practicability of such an operation, (no opportunity occurring of making the experiment on the subject previous to the removal of the eye from the orbit,) I substituted the eyes of various quadrupeds, and, in the presence of several medical friends, proceeded to its performance.

From these experiments, (for they were frequently repeated) such were the results obtained in establishing the facility with which the crystalline humour would admit of being removed from such a situation, that I had anticipated the pleasure of communicating to the profession, through the medium of your periodical publication, the particulars of an operation, possessing, on the one hand, all the advantages, and obviating, on the other, the following disadvantages of the usual mode of operation.

First, The objection arising out of the probability of the subsequent partial or total opacity of the cornea.

Secondly, The injury which frequently is occasioned to the iris, in the passage of the opaque lens, through its aperture or pupil into the anterior chamber.

Thirdly, The increased facility with which the divided parts are re-united, in consequence of the certainty with which the edges of the wound would remain in contact subsequently to the operation. And,

Fourthly, The possibility of thus removing, by extraction, the opaque crystalline lens, although union shall have previously taken place, betwixt the fibres of the iris whilst in a state of dilatation, and the capsule of the lens, the pupil being in such cases in a measure obliterated.

Notwithstanding, however, the favourable view which I had taken of this subject, in consequence of the nature of the the inferences deducible from my experiments, I determined, before giving them publicity, to pay further consideration, reflecting, that, as they had hitherto been confined to the eyes of oxen, sheep, &c. there might possibly exist some difference in the relative situation of the parts with respect to the human eye. Such a determination, I have experienced no reason for regretting, since, in opportunities which were shortly afterwards afforded me of making the same experiments on the eyes of the subject prior to the removal from the orbit, so different was the degree of facility with which the removal of the lens could even be accomplished, that I became somewhat discouraged from proceeding any further in my investigation.

In performing the operation on the subject, whilst the eye remained in the orbit, I made an incision through the schlerotic and choroid coats at a few lines distant from the edge of the transparent cornea, and occupying an extent of about two-thirds of an inch; with the extremity of the instrument having lacerated the capsule of the crystalline lens, the instrument was withdrawn, and a considerable degree of pressure made upon the globe of the eye, but without effecting the escape of the lens. Having found, that, in consequence of the toughness and inelasticity which the choroid and schlerotic coats possess, and their consequent incapability of yielding to the pressure of the lens from within in its endeavour to escape, it was necessary the incision should be enlarged; I increased in a very inconsiderable degree the extent of the incision, when, with the assistance of a very gentle pressure, the crystalline escaped; not alone, however, but unfortunately accompanied with a portion of the vitreous humour.

I have several times since repeated this experiment, and, from the sum of their results, have found ample and sufficient reason for the deduction of the inference, that, through an opening less in extent than is sufficient to admit of a partial protrusion of the vitreous humour, it is, in the generality of cases, impossible, without making use of a degree of pressure which would be incompatible with the subsequent maintenance of vision in the organ, to effect the escape of the opaque lens.

As the cause of its experiencing so much difficulty in its removal from this situation, through an incision less extensive, I can assign no other than the unyielding and unelastic property of the tunics, in connexion with the difficulty which the lens, being of a convex form, experiences, in common with all other convex bodies, in its passage through a more lineal aperture or section by incision. This latter circumstance cumstance I have endeavoured to remedy, by varying the form of the incision so as to render it more favourable to the convexity of the lens; such a variation of form, however, I have since found utterly impracticable, in consequence of the very inconsiderable space which exists betwixt the iris and the vitreous humour.

Finding such difficulties, therefore, in this operation wholly insurmountable, I had determined on not troubling you with any relation of my experiments, or even with its proposal; and, from such a resolution, should not have deviated, had I not, to my great surprise, on reading a recent number of the Edinburgh Medical and Surgical Journal, seen a communication from Dr. Von Embden, whence it appears that a discovery to the effect of this proposal had lately been made by Dr. Löbenstein Löbel, in consequence of an accidental occurrence. In concluding this paper, I have to observe, that its object is not that of depriving this gentleman of any merits he may himself attach to his discovery, but merely that of giving it as my opinion, that the operation of extracting the cataract thus conducted, will never be deserving of a preference to that in present use.

*Bath; March* 12, 1817.

